# Field Research Cooperative Wearable Systems: Challenges in Requirements, Design and Validation

**DOI:** 10.3390/s19204417

**Published:** 2019-10-12

**Authors:** Mateus C. Silva, Vicente J. P. Amorim, Sérvio P. Ribeiro, Ricardo A. R. Oliveira

**Affiliations:** 1 Department of Computing, Federal University of Ouro Preto, Morro do Cruzeiro Campus, Ouro Preto 35400-000, Brazil; vjpamorim@gmail.com (V.J.P.A.); rrabelo@gmail.com (R.A.R.O.); 2 Department of Biology, Federal University of Ouro Preto, Morro do Cruzeiro Campus, Ouro Preto 35400-000, Brazil; spribeiro@gmail.com

**Keywords:** cooperative wearable systems, field research, wearable device requirements, wearable systems design

## Abstract

The widespread availability of wearable devices is evolving them into cooperative systems. Communication and distribution aspects such as the Internet of Things, Wireless Body Area Networks, and Local Wireless Networks provide the means to develop multi-device platforms. Nevertheless, the field research environment presents a specific feature set, which increases the difficulty in the adoption of this technology. In this text, we review the main aspects of Field Research Gears and Wearable Devices. This review is made aiming to understand how to create cooperative systems based on wearable devices directed to the Field Research Context. For a better understanding, we developed a case study in which we propose a cooperative system architecture and provide validation aspects. For this matter, we provide an overview of a previous device architecture and study an integration proposal.

## 1. Introduction

Wearable devices are trending topics in different areas such as healthcare and activity recognition [1,2,3,4,5,6,7], sports [8,9], education [10,11], industry [12,13], human–computer interaction [14] and other areas. The rise of new concepts like the Internet of Things (IoT) [15,16,17] and Industry 4.0 [18] along with hardware miniaturization allow for the development of novel devices and solutions. Furthermore, the communication aspect on wearable systems is an important aspect on novel developed solutions [19,20].

Before studying the concepts involved in Field Research Cooperative Wearable Devices, it is crucial to understand the most significant features of this area. In addition, it is necessary to provide an overview of how wearable solutions are used in the studied context. Finally, we also need to present this work’s scope, objectives, and contributions.

### 1.1. Wearable Devices

An initial definition about the wearable device concept, provided by Rhodes [21], states that these elements are computers that are with the user all the time. Rhodes still affirms that wearable devices are portable while operating, and usable without blocking the hands. According to him, these devices have sensors, gather, and treat data even in a passive state, besides being always active and running.

Another feasible definition for wearable devices, presented by Bonato et al. [22], is that they are non-obtrusive devices. In other words, wearable equipment cannot obstruct the user’s movements during usage. Furthermore, Billinghurst and Starner [23] state that wearable systems must satisfy three different objectives:

1. They must present mobility;
2. They must augment reality;
3. They must add context-awareness.

Pascoe [24] states that wearable devices’ context-awareness comes from embedded sensors. These gadgets should gather data, adapt, find resources, and augment reality. Wei et al. [25] discuss that, in modern approaches, these elements also have connectivity. For instance, this allows the integration with applications using cloud computing and an intersection with the IoT.

### 1.2. Cases of Use: Outdoors and Field Research Wearable Devices

In the last subsection, we presented wearable devices’ principal concepts. Here, we provide an overview of the propositions and use cases from field research applications found in the literature. This information is essential to understand the features and validation processes applied to these solutions and how they will further relate to our case-study.

Delabrida et al. [26] developed a Head-Up Display (HUD) to provide useful information in ecological field research processes. Their solution aims to help geologists in some of their in-field measurement activities. In addition, their solution monitors environmental safety information, such as weather conditions. To validate their appliance, they tested the algorithm accuracy and system performance with different boards.

Silva et al. [27] developed a Smart-Helmet for field research processes in ecology. Their device objective is to help ecologists performing canopy studies on the forest environment. To do so, they collect distance information, orientation, and images from the environment. They validate their appliance testing the energy consumption, sensors’ data quality, and transmission quality.

Silva et al. [28] also evolved their Smart-Helmet solution for field research in ecology. Again, this solution aims to help ecologists performing forest canopy studies. To validate their system, they provided a validation routine for their data-fusion and augmented reality algorithm.

Biremboin et al. [29] present a system based on commercial off-the-shelf wearable devices to monitor the user’s mental state. With this solution, they aim to monitor the user during outdoor activities. To validate their system, they performed a test with different users walking in a predefined outdoor route in Utrecht, the Netherlands.

Delabrida et al. [30] proposed a wearable system for 3D data acquisition and environment reconstruction. This proposal also aims to help ecologists performing canopy studies, reproducing the forest in a virtual environment. Their proposal presents the main architectural features and identifies the most critical flaw sources. Nevertheless, they do not use a formal practical validation process.

Delabrida et al. [31] developed a Head-Mounted Display (HMD) to help geologists. This proposal aims to help these researchers in common in-field measurement activities, providing a visualization using Augmented Reality. To validate aspects of this solution, they performed an Operating Systems (OS) evaluation, an algorithm performance evaluation, cross-platform hardware evaluation, and an energy consumption evaluation.

Chen et al. [32] presented a wearable device to detect heat strokes. This solution uses sensors to detect body temperature, heart rate, GPS location, and environmental conditions to determine the risk of heatstroke. To validate their system, they developed an experimental condition in which they measured vital signals of a male subject with the proposed prototype.

Velazquez et al. [33] propose a novel wearable device to aid visually impaired users in outdoor navigation. Their tool uses GPS and tactile feedback signs to help to indicate directions in the user’s shoe. They validated their prototype with early tests of the system performance.

These research works propose and validate devices or systems applied in field research or outdoor environments. Moreover, they target health hazards, dangerous activities, or difficulties due to impairments. In every case, they examine their case of use analyzing pieces of information about the process. Afterwards, these researchers propose devices or systems to extract the information based on wearable sensors and even provide some feedback to the user. Finally, most of them validate their systems using tests based on the identified system requirements.

### 1.3. Objective

In the last two subsections, we presented the concepts around wearable devices and systems. In addition, we displayed some use cases where these concepts apply in field research. In the former subsection, we mainly analyzed how the area researchers proposed and validated systems based on wearable devices in different appliances and concepts. Here, we explain the main objectives of this work.

In a first analysis, we could not find the usage of cooperative wearable systems in field research. Although some of the research present devices with distributed computer nodes working in cooperation, none of them explore the integration of multiple wearable devices in this context. Thus, this work has the following main objective:

- A study of the challenges in the requirement establishment, design, and validation processes for Field Research Cooperative Wearable Systems.

We perform this study through two steps. The first one is a theoretical approach, reviewing the state-of-the-art articles on the area, and presenting how the researchers systematically approach the requirement definition, design process, and validation. The second one is a practical case-study, in which we apply this systematical approach to validate and analyze these methods.

In the first stage, we overview the requirement establishment and design process for Field Research Cooperative Wearable Systems. From our presented review, we understand that this perspective adds flexibility and modularity to wearable systems design. In addition, we present a general approach to the design and validation method applied by the researchers in the latest articles found in the literature.

In the second stage, we also want to validate the requirement establishment and design process through a case-study. This validation follows the reviewed and presented methods. For this matter, we develop a platform based on a previously verified platform, proposed by Amorim et al. [34]. This process focuses on the design of a novel system architecture using validated devices to the detriment of novel device architecture. This proposal follows an organization called the Multiple-User Cooperative Wearable System.

### 1.4. Contributions

In this paper, we provide two main stages to approach the main objective. The first one is a theoretical analysis of the requirements, design aspects and possible architectures for Cooperative Wearable Systems and field research devices. The second one is a case-study using the theoretical review to approach a solution. Thus, we summarize our main contributions as:

- A theoretical analysis of the requirement establishment process of field research gear;
- A theoretical approach to the wearable system design process stages;
- A conceptualization from Cooperative Wearable Systems (CWS), especially applied to the field research context (FR-CWS);
- A case-study validation using the Multiple-User Cooperative Wearable Systems concepts, with:
  - A context overview, containing some related applications;
  - A requirement estimation based on the context analysis and regarding the challenges identified in the theoretical overview;
  - A novel FR-CWS architecture proposal, considering the information gathered on the conceptualization stage;
  - A formal validation testbed, considering ground aspects from this kind of device;
- A discussion, formalizing the main challenges identified through both stages.

### 1.5. Text Organization

For the proposed matter, Section 1 introduces the concepts of wearable devices and presents some cases of use in outdoor and field applications. In Section 2, we define the boundaries of field research gear and how to establish their requirements. Furthermore, we overview the wearable systems design process and conceptualize Cooperative Wearable Systems. Section 3 presents this text’s case study steps. It introduces the requirements, device architecture, system architecture, evaluation methods, and results. Finally, we discuss the obtained theoretical and practical results in Section 4.

## 2. Conceptualization

In the previous section, we presented the main concepts around wearable devices, as well as some of the applications in similar contexts to this work. This section presents an overview and helps to create the main concepts around Field Research Cooperative Wearable Systems (FR-CWS). At first, we need to understand the requirements of field research equipment. Afterwards, we need to overview aspects of wearable systems design. Furthermore, we need to review existing cooperative wearable systems, understanding the involved technologies and concepts.

### 2.1. Field Research Equipment Requirements

An essential stage in any field research is the equipment selection. Nevertheless, this task is not simple. For instance, Shapira and Goldberg [35] affirm that this is a key factor for the success of a construction project. In addition, Zaneldin and Sivaloganathan [36] state that the process of selecting suitable equipment in this context is essential in heavy industrial processes.

Similarly, every field research process has its occupational hazards. Yilmaz Kaya and Dağdeviren [37] affirm that the equipment selection may follow a universal design (UD) pattern. The UD principles are:

- Equitable Use;
- Flexibility;
- Simple and intuitive use;
- Provide perceptible information;
- Error Tolerance;
- Low physical effort;
- Appropriate dimensions.

In [35,37], the researchers also state that the safety equipment selection follows an analytic hierarchic process (AHP). This process identifies the main targeted problem, along with its factors and subfactors.

Therefore, when proposing novel wearable solutions for field research with occupational hazards, a suitable outcome is to adapt new functionalities onto previously selected gear, and then to implement and try to adopt different external modules. This process follows both the UD pattern principles without the need for new AHPs.

### 2.2. Wearable Systems Design

The wearables creation process commonly goes through a set of steps aimed at defining each of their internal components, whether related to hardware or software. It is common that, during the process of building these devices, their organization/architecture will be approached at some point, commonly in the early stages. Often, each new device being developed eventually restarts the creation process that goes through all the steps again, without reusing any already created part.

Then, creating wearable devices that can be reused requires standardizing the whole construction process. These hardware and software components’ organization/architecture standardization is an early and necessary stage in the wearable devices’ development process. A standardization may pave the way to potentially reuse the same solution in a variety of applications context. This subsection presents a proposed methodology for building wearable prototypes/products. Steps presented in this process can be thoroughly carried out “as is”, or have some parts modified/removed as a way to comply with the desired specification.

The last subsection presented design aspects and the equipment selection process for field research tasks. This section presents an analysis of some factors of the wearable devices design process and general methodology. This is important to understand the most relevant variables when proposing novel tools and architectures. This paper analyzes hardware and software project practices and the usual methodologies employed during the design process of wearable systems [34].

**Figure 1:**
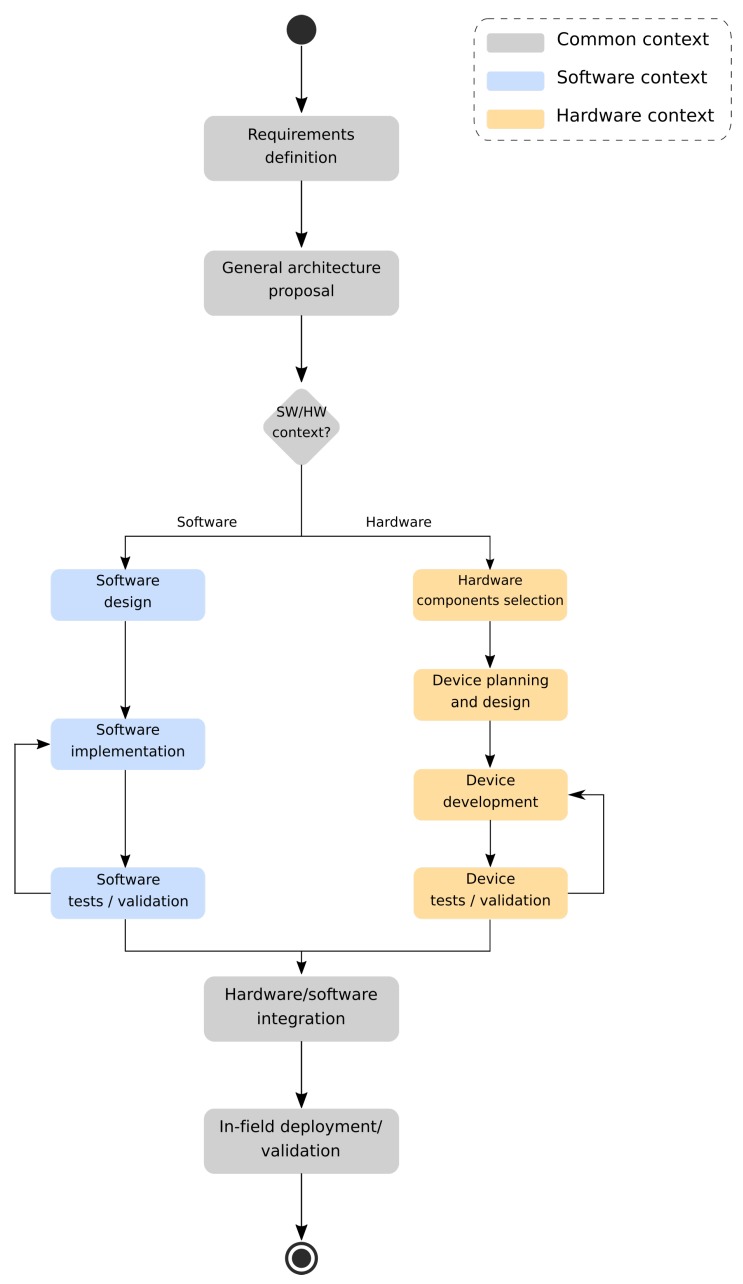
Diagram outlining the steps taken into consideration when designing a new wearable solution.

A general analysis of wearable devices design process can proceed considering the two main macroblocks: hardware and software parts. However, there is a common core that runs through both contexts. Figure 1 presents a general overview of common steps usually taken into consideration when developing wearables research. It also covers standard context blocks and specific ones related to software or hardware parts. Additionally, it is also outlined that hardware and software designs share specific modules:

- **Requirements definition:** Step of requirements gathering through the final application context analysis. Data collected here influence the whole process until the wearable solution deployment; and
- **General architecture proposal:** Requirements work as inputs to create a general architecture, enclosing hardware and software parts. The architecture proposal also allows for figuring out how hardware and software parts will communicate with each other.

However, besides these two initial modules, there are specific parts that will be carried out only in one of these contexts of development.

- **Hardware:** Commonly used methodology to plan, design and build the wearable prototype hardware part.
  - **Hardware components selection:** After requirements and architecture definition, it is possible to enumerate the necessary hardware components/modules that will support the desired features;
  - **Prototype planning and design:** Hardware components serve as input data to plan and design the final prototype. In this step, components connections are defined, and Printed Circuit Boards (PCB) layout is outlined, if necessary;
  - **Prototype development:** This part will carry out wearable device manufacturing. Main Central Processing Unit (CPU) and hardware peripheral components will be physically connected, possibly using a final version of PCBs designed in the previous step. In the end, components can be attached to the garment; and
  - **Device tests/validation:** Several rounds of in-place hardware tests and validation to verify the connections made with each component. If this part does not work as specified or pass the validation tests, specific points can be fixed/changed in a new development round.
- **Software:** Considered methodology when developing a wearable prototype associated software solution.
  - **Software design:** The previously specified requirements can also be used as a reference to design the related software. This step involves functionalities definition, as well as the planning of how inner modules will interact with each other;
  - **Software implementation:** Design planned before may now be used as a reference to properly code the software. Commonly, specific and lightweight programming languages/frameworks can be used here, according to the whole solution needs;
  - **Software tests/validation:** This module is in charge to test and check if the resulting software is compliant with previously specified requirements. If the solution does not fulfill the requirements (or does not pass the validation tests), a new development round can be carried out.

The remaining blocks are used by both contexts:

- **Hardware/software integration:** This part encloses the integration of previously developed hardware and software parts. This is a mandatory step, once both of these parts were independently developed until here. Basic and composed functionalities can be evaluated, verifying whether they provide the expected output or not; and
- **In-field deployment/validation:** This is the final step of wearable systems design. The wearable device, which now has integrated hardware and software modules, are deployed and validated during in-field sessions. This occasion is also used in several research projects as the moment to gather and retrieve empirical data.

The design process shown here is sometimes also used with some additions. The research presented by Liang et al. [38] extends the diagram presented in Figure 1, adding a new software part in a cloud-centered infrastructure. Indeed, Lim et al. [39] address the system design and development in a simplified way. Despite this simplification, all elements presented here are still available in this research.

### 2.3. Cooperative Wearable Systems

After comprehending the design process for wearable devices, we also need to understand how wearable devices and systems are used in cooperative systems and environments. Zheng et al. [40] reaffirm that this integration brings innovations to different areas such as healthcare, sports, entertainment, and others. In some cases, the acquired data can even be integrated into cloud applications, as presented by Fortino et al. [41]. Thus, it is also vital to understand the architecture proposal and validation processes.

Xu et al. [42] state that wearable devices produce a large amount of information, which can provide useful resources for external algorithms that process this data. Ometov et al. [43] enforce the idea that cooperative wearable applications have some design challenges, especially related to heterogeneous communication and offloaded computation. In addition, Zhang et al. [44] affirm that communication delays are also a cost factor in IoT cooperative networks. They enforce that minimizing energy and delay costs is crucial in this context. Furthermore, an optimization method as the one proposed by these researchers helps to identify sensed events and even cooperative decisions in architectures as the ones evaluated in this work.

Augimeri et al. [45] and Fortino et al. [46] present four different conjectures for collaborative body sensors. In two of them, the data gathered from a single user provide data to one or multiple stations. In the other two, data gathered from multiple users feeds one or multiple stations. Similarly, to understand the ways to cooperate using wearable devices, we categorize the Cooperative Wearable Systems into two scenarios:

1. Single-User Cooperative Wearable Systems,
2. Multiple-User Cooperative Wearable Systems.

The first type happens when the same user utilizes multiple wearable devices to compose a system, feeding one or multiple applications in base stations. The second type occurs when many users wear the same equipment, with post-processed data and flexibility gained in the final appliances in individual or various consumer stations.

#### 2.3.1. Single-User Cooperative Wearable Systems

Wearable compositions are important to monitor the context of the environment and also the user. Mihovska and Sarkar [47] introduce an important concept for this context: Human-Centric Sensing. According to these researchers, the IoT and connectivity of modern devices provide the tools to create cooperative systems around the user. These appliances can monitor both user signs and the surrounding environment.

Zhang et al. [48] proposed a cooperative environment based on a glove-shaped pressure sensor and an armband. This system was designed to provide gesture recognition for Human–Computer Interaction devices. To validate this system, they performed a set of tests to verify the recognition accuracy.

Peng and Peng [49] affirm that Body-Area Networks became an important tool to create novel healthcare solutions with collaborative wearable devices. They proposed a cooperative communication strategy to integrate multiple devices in a Wireless Body-Area Network (WBAN). They validated their proposal using simulations.

Nguyen-Huu et al. [50] present a combination of a wearable device and smartphone working in cooperation to monitor daily activities in an indoor environment. Their system uses both an armband and a smartphone to gather sensor data, process and recognize activities, and transmit this information to a web server for further analysis. They validated their system using performance evaluations on their activity recognition and lifelogging algorithms.

Most of the proposed systems and architectures use wearable devices as IoT nodes in a Wireless Body-Area Network. In some situations, they also employ smartphones as connection gateways. Finally, these works follow the same systematical process, starting with requirement analysis, architecture proposal, implementation, and validation.

#### 2.3.2. Multiple-User Cooperative Wearable Systems

Pimentel et al. [51] present a multiple-wearable cooperation system for monitoring the stress of multiple surgeons. They use commercial devices for vital signs monitoring, gathering ECG and actigraphy signals. An Android Application gathers the data from the wearable devices, marks events, creates reports, and stores this data in a database. Finally, they used several statistical studies to validate the self-assessment tools present in the Android application.

Prakash and Ganesh [52] established a wearable-based communication environment for cooperative health monitoring in hospitals. They interpret this environment as a Wireless Sensor Network (WSN). To validate this environment, they used network simulator applications and verified the packet transmission performance.

Pham et al. [53] present and validate a wearable-based system environment to monitor older adults and patients with Parkinson’s disease. They propose an architecture based on IoT wearable devices with Inertial Measurement Units (IMUs) located on the user’s lower limb. From the data of this device, the system tries to recognize postural transitions. To validate this proposal, they tested their environment and system with actual target users with Parkinson’s Disease and older adults, and compared the results with video recognition, using statistical analysis.

Even in a multiple-user context, the state-of-the-art works follow the same systematical process in the systems proposal. They analyze the context of use and gather the system requirements, propose an architecture, assemble a prototype environment to test their proposal, and perform validation tests.

## 3. Case Study

In Section 1, we presented the definitions around wearable devices and their cases of use in field research. In Section 2, we built and reviewed the concepts encompassing Field Research Cooperative Wearable Systems (FR-CWS). For this matter, we understood the requirement verification and design processes, proposing the concept of Cooperative Wearable Systems (CWS).

**Figure 2:**
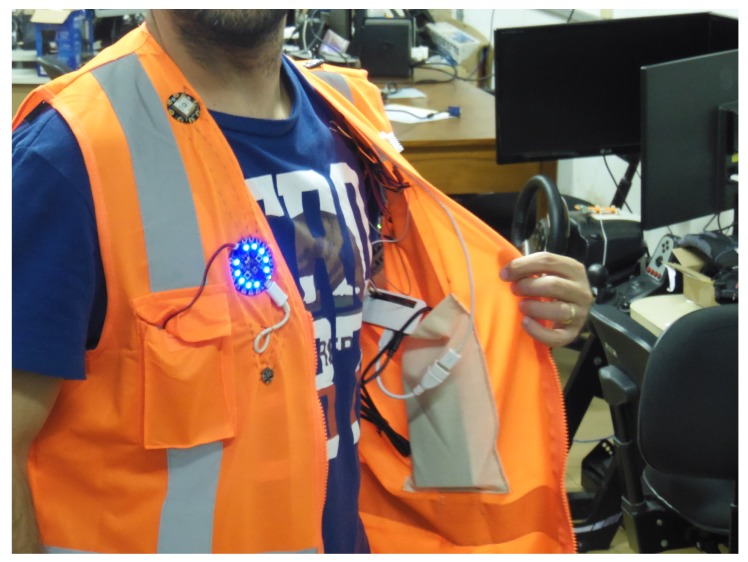
Wearable Device Prototype, proposed in [34].

In this section, we perform a case-study to validate the presented processes. This system is based on a prototype proposed by Amorim et al. [34], displayed in Figure 2. We do not propose any change in the hardware configuration since our objective here is not to validate the device alone, but the application of multiple devices. All sensors were previously used side-by-side, and tested during the prototype development stage.

We explore the fact that this solution was previously proposed and validated. As presented in Section 2.1, the proposal of novel devices requires a systematic process. For this matter, we follow the concept of Multiple-User Cooperative Wearable Systems, presented in Section 2.3.2, exploring the usage of the same device in different applications.

Hence, we use the knowledge of the present sensors to describe novel applications that can use this data to generate new information. This study analyzes the evolution of this system towards a CWS, and, more specifically, a FR-CWS. This process follows the observed methods for the proposal and validation of wearable devices and systems. Furthermore, we create a conjecture in which this system can attend different appliances.

### 3.1. Context Overview

This subsection presents the context in which we apply the proposed solution. First, our wearable device collects useful information from the user and the surrounding environment. At first, this wearable was designed to support workers in open-sky mining industry [34]. Thus, this work considers a broader hypothetical scenario in forest field research. It was created based on usual tasks from wearable devices and field research. In such a scenario, a multidisciplinary team gathers different information from the device. The members of this team are:

1. A medic, monitoring the team’s health [54,55];
2. A biologist/ecologist, measuring sensor values for his research [56,57,58];
3. A physiologist, studying the physical effort in each kind of task [59,60,61];
4. A navigator, cross-checking the global position with physical or virtual maps [33,62,63].

Each of the members of this group wears the same safety field research gear and has access to the data of the whole team gear. In addition, each member has access to a Smartphone app with specifically-designed features to their professional functions. This architecture seeks to use the flexibility of the device produced data to attend different subjects with the same wearable, as a characteristic of multiple-user CWS.

### 3.2. Requirements

This subsection presents an overview of the requirements for the context of this wearable system. The first set of specifications come from the wearable devices common features:

- The device must not block the user’s common movements;
- It must provide information from sensors;
- It must detect context changes.

Furthermore, as presented in Section 2, the adoption of new devices in field research require the creation of novel AHPs. Thus, it is better to adapt the wearable system into common-use safety gear. The adoption of these devices prevents the process of implanting a new safety device. Hence, as a case study, we chose a safety vest as the baseline from our proposed solution.

Henceforth, the team will be identified as M for the medic, E for the Ecologist, P for the Physiologist and N for the navigator. For the demands of the multidisciplinary team, this device must have sensors which provide:

- Body temperature and humidity (M, P);
- Heart rate and blood oxygenation (M, P);
- Environmental luminosity, temperature and humidity (M, P, E);
- Global Position and Altitude (E, N);
- Muscular Effort (P);
- Body Motion (M, P);
- Safety lights (M, E, N, P).

Each professional can obtain the required information from all the crew members, in an application designed with the specifications from each of them. This feature comes with the flexibility of CWS.

### 3.3. Device Architecture Description

**Figure 3:**
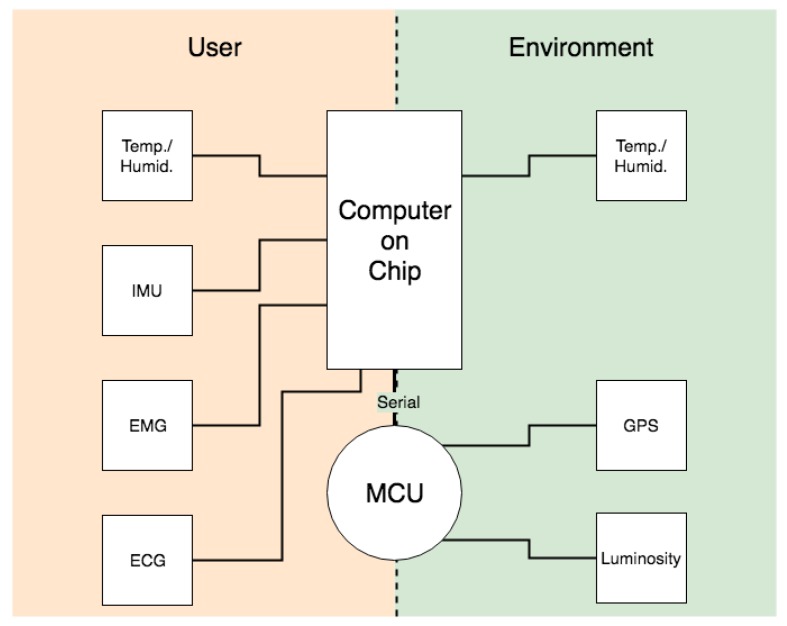
Proposed device architecture.

In the last subsection, we presented the requirements for the proposed CWS. In this subsection, we present the architecture and features of the prototype applied in this context. Figure 3 represents the main elements of this prototype.

As mentioned, this device was previously presented by Amorim et al. [34]. It has sensors to monitor both the user and some environmental variables. These sensors are:

- User Sensors:
  - **Temperature/Humidity Sensor**—This sensor monitors the temperature and humidity internally. This sensor connects reading digital data from a GPIO pin;
  - **IMU Sensor**—This sensor monitors the user’s body motion. It communicates using the I2C bus;
  - **EMG Sensor**—This sensor monitors the muscular effort from the user. It transmits the measured data using GPIO monitoring;
  - **ECG Sensor**—This sensor monitors the heart rate and blood oxygenation. It communicates using the I2C bus.
- Environmental Sensors:
  - **Temperature/Humidity Sensor**—This sensor monitors the temperature and humidity externally. This sensor connects reading digital data from a GPIO pin;
  - **GPS Sensor**—This sensor gathers the global position data and transmits it to the computer board through the MCU. The MCU uses a serial connection to communicate with this board;
  - **Luminosity Sensor** - This sensor gathers luminosity data and transmits it to the computer board through the MCU. The MCU uses I2C connection to communicate with this sensor.

**Figure 4:**
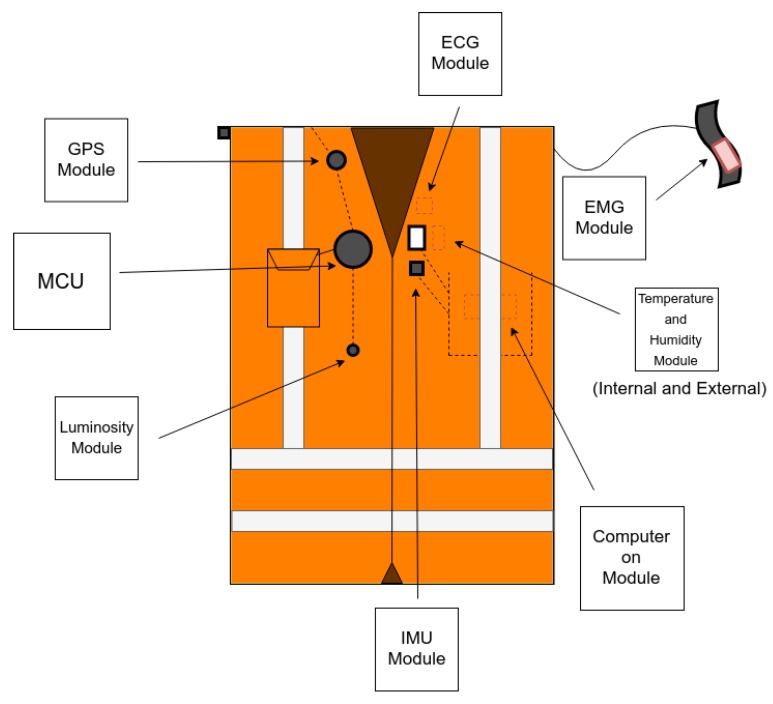
Wearable device illustration.

Furthermore, the vest provides the option of actuation. The MCU can use the luminosity sensor to activate safety lights in case it detects a dark environment. For a better understanding of the organization of this device sensors, Figure 4 presents an illustration of the components’ geometrical location. As presented at the beginning of this section, this device was previously tested and validated in [34]. All of the elements fit on a safety vest and don’t block or bother the user.

For a broader comprehension of the system’s capabilities, it is also important to map the data acquisition time for each sensor. This information also helps to create the simulated devices in the validation stage. For this matter, we summarized the acquisition time for each sensor present in the prototype developed by Amorim et al. [34]. Table 1 presents this summary. In addition, we consider that the consuming applications will further post-process the sensors acquired data. Therefore, the presented sampling times consider the smallest interval required to acquire this minimal information.

This analysis used the provided information from the sensors and the computer-on-chip datasheets [64,65,66,67,68,69]. Finally, we consider that all sensors were properly calibrated in a previous assembly stage with the correct methods. For a broader comprehension, we also present the general characteristic of the calibration process for each sensor. The AM2302 sensor needs a chemical process in a closed chamber for calibration, as it is an hygrometer–thermometer [70]. The MPU-6050 is a 6-DoF IMU, which requires a 3-axis motion and spinning movements [71]. As an EMG sensor, Myoware must be calibrated before the usage, considering reference levels of contraction signals [72]. As a GPS module, FGPMMOPA6H requires a factory calibration according to its antenna [73]. TSL2561 is a lux meter, and therefore must also be previously calibrated with its response curve [74]. Finally, MAX30100 is a pulse-oximeter, and thus requires factory calibration with the light wavelength response [75].

### 3.4. System Architecture

In the last subsection, we presented the main features of the wearable devices which will compose this system. In this subsection, we present the proposed CWS architecture. We based the architecture in the multidisciplinary team presented in Section 3.1. As mentioned, the crew is composed by a medic (M), an ecologist (E), a physiologist (P), and a navigator (N).

This system follows the concept of Multiple-User CWS, defined in Section 2. Within this context, multiple users wear the same device. The flexibility gain happens in the application level, where the data collected from the wearable devices will be post-processed.

**Figure 5:**
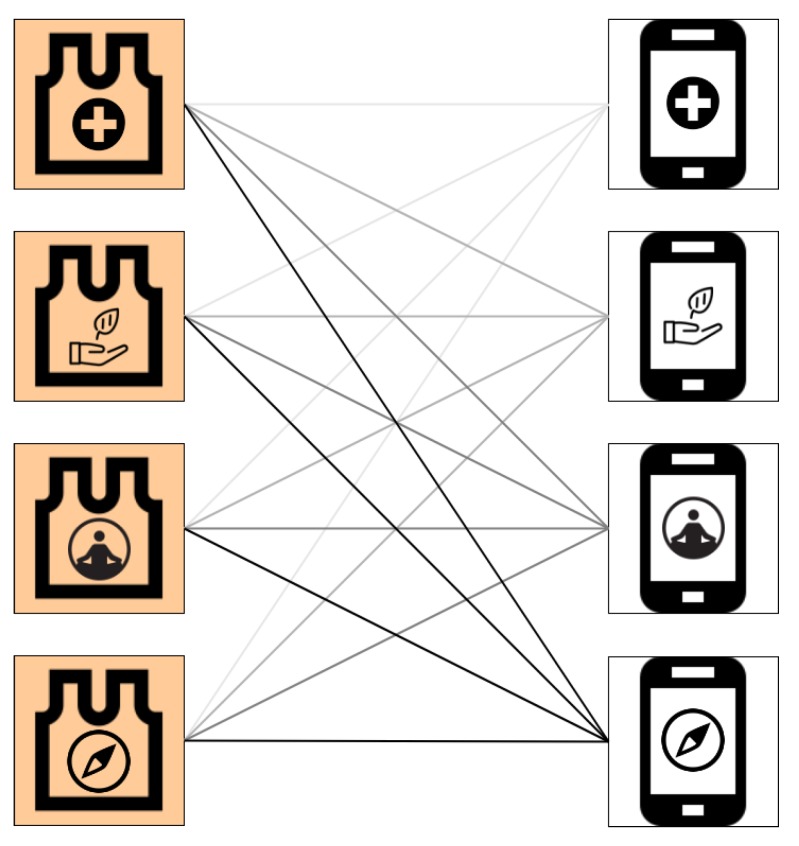
Field research cooperative wearable system architecture.

Figure 5 represents the CWS architecture integration. As mentioned, each crew member has an application to gather the sensors’ data using wireless communication protocols. From the complete data, the applications separate useful information for each professional involved in the process.

In modern applications, wearable devices can be interpreted as IoT-nodes [76,77,78,79,80,81]. Thus, in the context of this work, each wearable is also modeled as an IoT-node composing a FR-CWS architecture. Each member of the crew can use a smartphone connected to the network or to a gateway to query the data from each wearable device.

Each crew member application has a proper sampling interval need, according to each professional specialty. Nonetheless, this sampling rate must consider the sensor reading interval and communication latency, natural in this kind of system. Before moving forward, it is also important to understand the communication options for composing a wireless network. Mahmoud and Mohamad [82] divide the network protocols for the IoT according to the communication range, following this nomenclature:

- Proximity (up to 10 m);
- Wireless Personal Area Network (WPAN) (up to 100 m);
- Wireless Local Area Network (WLAN) (Up to 1000 m);
- Wireless Neighborhood Area Network (WNAN) (up to 10 km);
- Wireless Wide Area Network (WWAN) (up to 100 km).

According to the conjectured scenario, we desire that the users have access to each other data in distances within the ranges of WLAN and WPAN. For this matter, this work also provides some examples of technologies in use in these scenarios. Table 2 presents these options.

In a test scenario, we use the WLAN as connection mean, as it is enough to manage the information exchange for a crew working in proximity.

### 3.5. Evaluation Methods

As the proposed system is a distributed device network, an important aspect of the architecture is its communication restraints. To validate this solution as a cooperative multiple-node system, we need to analyze the feasibility of the proposed system. In addition, we need to understand the limitations of the data availability in this network before any algorithmic proposal. Thus, for this evaluation, we define a mathematical model based on Quality-of-Service (QoS), like the ones presented by Boukerche and Samarah [83] and Silva and Oliveira [84]. This QoS test evaluates the information availability, providing the temporal constraints that must be evaluated when creating consuming applications.

At first, we assume that the transmission time is divided in equally-sized timeslots, represented by the set $T=\{t_{1},t_{2},t_{3},\ldots ,t_{m}\}$, where $t_{i+1}-t_{i}=\lambda$ for 1<i≤m.

**Definition** **1.** *Let $D=\{d_{1},d_{2},d_{3},\ldots ,d_{n}\}$ be the set of n wearable devices present in the network.*

**Definition** **2.** *Let $p_{j}=d_{r}\ldots d_{s}$ be a data acquisition pattern, where each $d_{i}$ element is a device from D ($d_{i}\in D$).*

**Definition** **3.** *Let $P=\{p_{1},p_{2},p_{3},\ldots ,p_{k}\}$ be a set of k desired observation patterns.*

**Definition** **4.** *Let $F(p_{a},x)$ be the number of observations of a $p_{a}$ pattern within an x number of timeslots.*

**Definition** **5.** *Let $F(p_{a})$ be the total number of observations of a $p_{a}$ pattern in the whole test period.*

The quality parameter $Q_{s}\left(p_{i},k\right)$ for a pattern $p_{i}\in P$ expected in a *k* number of timeslots is defined by the following equation:

(1) $$Q_{s}\left(p_{i},k\right)=\frac{F(p_{i},k)}{F(p_{i})}.$$

In our scenario, each wearable will be simulated using a Raspberry Pi Zero W computer-on-chip connected to the network. These devices were chosen as they were the main hardware nodes in the initial prototype developed by Amorim et al. [34].

In every possible scenario, the professionals may want to receive the messages from one member, two members, three members or the whole crew. If we consider our pattern set *P* defined by all the permutations of arrangements from the elements of *D* according to this rule, our complete pattern set has a size *S* of:

$$\begin{array}{c} S=P(4,1)+P(4,2)+P(4,3)+P(4,4),\\ S=\frac{4!}{3!}+\frac{4!}{2!}+\frac{4!}{1!}+\frac{4!}{0!},\\ S=64. \end{array}$$

Thus, for each possible combination of *P*, we performed 30 tests for each device running in parallel, trying to obtain the same data. On each test, the consumer applications concurrently try to retrieve the data from each sensor according to the possible message patterns of *P*. Each consumer application is generated as a generic client in the network, with the capability of establishing a connection with each node through the network gateway and querying its sensor data. In addition, in each test, the consumer applications will try to gather the same data. Finally, the wearable devices will be available during the whole process, as a wearable and IoT requirement.

After running the tests, we analyze the data to check the quality factors defined according to the timing requirements presented in Section 3.4. For simulation purposes, the string containing the simulated data will be released after an interval of 4.2 s.

### 3.6. Results

In the last subsection, we presented the validation test set for the proposed case-study. Here, we analyze the created modules and practical aspects of the realized tests. The proposed test set is a simulation of the actual network environment. For this matter, we developed a server application in four different computer-on-chip nodes, connected to a WLAN network, as mentioned in Section 3.5. In addition, we configured four different computers as clients to query data from the server nodes.

The client nodes can perform one query at a time. This avoids one application to block the sensor nodes after connecting. As mentioned above, each client application runs the same code, as well as each sensor node executing the same server routine. The devices are distributed in the local wireless network as presented in Figure 5.

In Section 3.5, we also mentioned that the test covers each message scenario of possible queries. The evaluation method considers the Equation (1), presenting the quality factor for a time window of *k*-timeslots. As presented in Table 1 and Section 3.5, the total time to obtain the data from all sensors is around 4.2 s. Thus, we decided to analyze the discrete-time slots in intervals of λ=1 s. In addition, the quality factor must consider the number of queries in a pattern, as each query will take at least five timeslots to be solved. Therefore, after deciding the *k* factor, the analysis uses the following equation to calculate the actual $k_{l}$ factor, where *n* is the number of queries in the $p_{j}$ pattern, $p_{j}\in P$:

$$k_{l}=n.k.$$

In addition, as presented before, there are a total of 64 unique patterns considering the single combinations of each $d_{i}$ device, $d_{i}\in D$. Therefore, we collected the times for obtaining each pattern $p_{j}\in P$ composed of a unique combination of devices.

**Figure 6:**
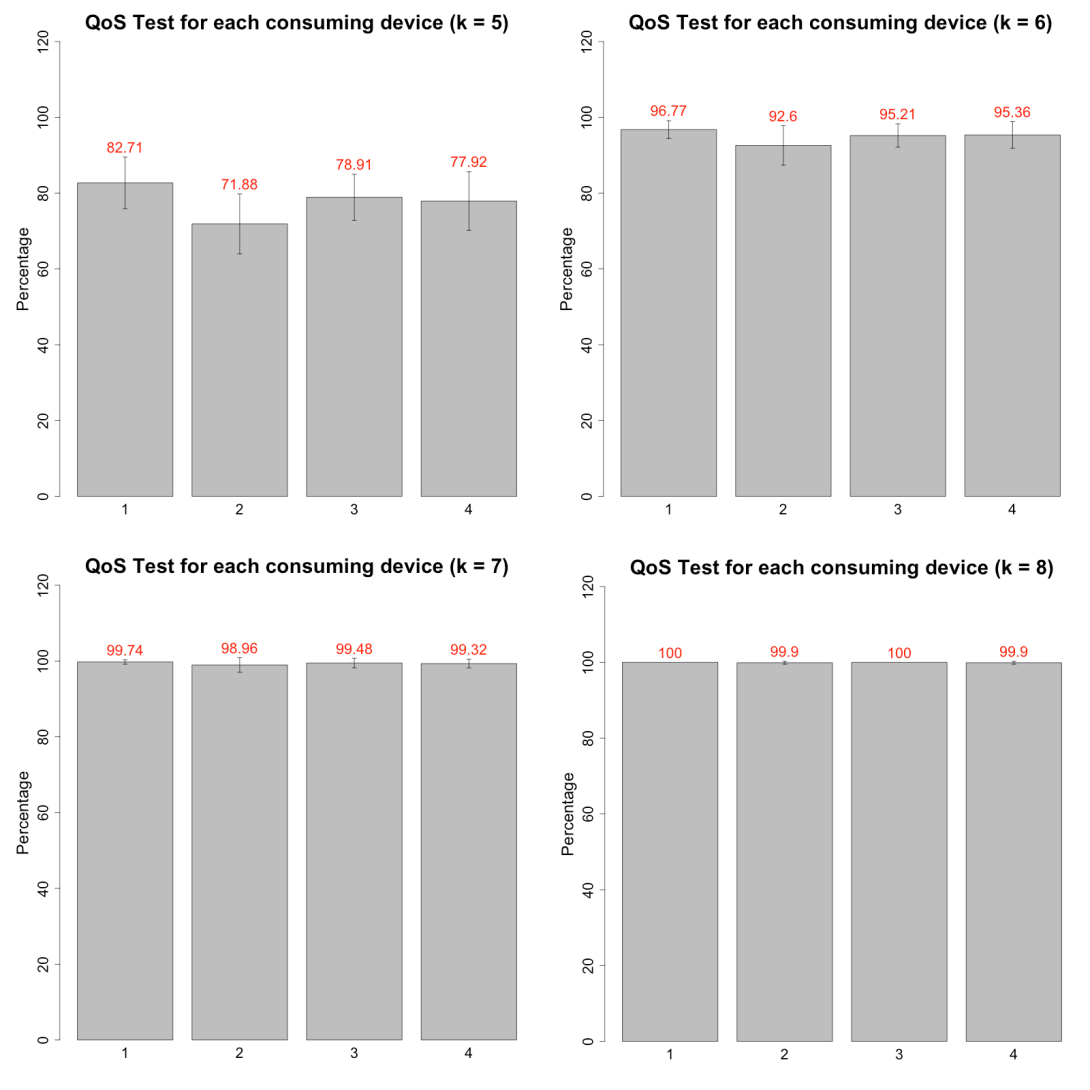
Results of the QoS tests on each device.

All four client devices repeated this procedure 30 times, executing in parallel and concurrently querying data from the server nodes. Finally, we analyzed the data considering different *k* values, starting with k=5. Figure 6 displays the obtained results from the tests.

The objective of this analysis is also to understand the constraints of the proposed architecture and its elements. The QoS factor is a fraction of the total observations of a pattern. Therefore, we present the results as percentages.

The results of the QoS tests indicate that the minimum number of timeslots to guarantee the complete delivery of patterns is nine slots, or in other words, nine seconds. As mentioned, the minimum number of slots necessary to generate and transmit the data are five. Thus, the applications of each research must consider using acquisition rates between 5 and 9 seconds per device.

**Figure 7:**
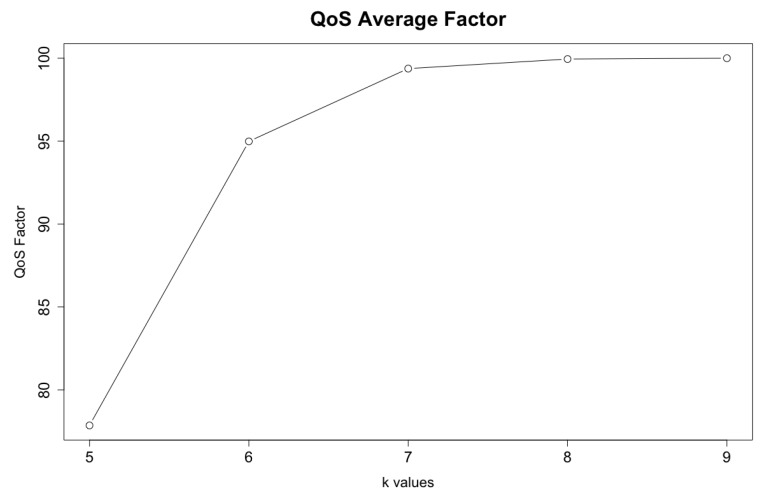
QoS average factor for each *k* value.

The results show that the average QoS factor for k=5 is 77.8%. The average QoS factor for k=6 is 95.0%. The average QoS factor for k=7 is 99.4%. The average QoS factor for k=8 is 99.9%. The quality factor is 100% for k=9. As expected, the lower the value of *k* used in the analysis, the worse the quality factor result. This happens due to sensors’ acquisition latency and network concurrency. Figure 7 displays the average factor trend for each *k* value.

Another conclusion from this result is that the gain is small starting from k=7. Increasing the number of timeslots from k=5 to k=6 elevates the average quality factor by 17.1%. Increasing from k=6 to k=7 increases the quality factor in 4.4%. Increasing from k=7 to k=8 raises the quality factor only by 0.5%. Finally, increasing from k=8 to k=9 only elevates the quality factor by 0.1%.

The first conclusion with these test results is the validation of the network architecture. The test displays the capability of concurrently querying the wearable node devices for messages in an IoT-like application using wearable devices, even with the sensor reading expected latency. This result confirms the feasibility of the creation of a Field Research Cooperative Wearable System in the context of the case study. Furthermore, the creation of applications to consume the data from the wearable nodes should consider the timing constraints indicated by the QoS test. On average, the devices must plan to have an acquisition rate of around 7 s for each node. Finally, in the scope of this text, the usage of a 7 s acquisition rate guarantees a QoS factor of 99.4%.

## 4. Conclusions and Discussion

In this text, we presented both a theoretical and a practical analysis around Field Research Cooperative Wearable Systems (FR-CWS). At first, we presented the main context around wearable devices and some cases of use from wearable devices in Outdoor and Field Research applications.

In the following section, we reviewed and presented the main concepts of our theoretical analysis. We analyzed the requirement gathering process, as well as the wearable systems design procedure. Furthermore, we proposed the concept of Cooperative Wearable Systems (CWS), in single and multiple user applications.

The third main part of this work is a case-study method of designing and developing a cooperative wearable system for a field research appliance. We follow the design pattern proposed in Section 2, defining requirements, proposing an architecture and developing a Hardware and Software implementation to test aspects of the proposed architecture.

### 4.1. Requirements of Field Research Devices

In Section 2.1, we analyzed the main aspects around field research gear requirements. An important aspect of this matter is the definition of the correct equipment to use. This factor is vital to the success of the project. In addition, the gear selection must consider universal design principles and a hierarchic process. Thus, it is better to use the previously approved gear as a baseline to create novel solutions.

### 4.2. Design Aspects for Field Research Cooperative Wearable Systems

The wearable system design pattern follows a hardware and software co-design process. This procedure starts with the requirement definition for the problem, analyzing the solution context. From this stage, the creators propose a general architecture, segmenting the elements into hardware and software parts. These parts are designed in parallel and integrated into the main solution. Finally, the designers validate aspects of the proposed solutions with methodical tests.

### 4.3. Field Research Device Design and Validation

We applied the proposed methodical design pattern in a case-study. This task aims to analyze the proposed method in the context of a CWS, and more specifically in FR-CWS, where the constraints are firmer. This process also starts with a requirement gathering, considering information from the desired task. Starting from the Multiple-User CWS concept, this system proposes to use multiple versions of the same device to feed multiple applications with different objectives.

We started using the device proposed by Amorim et al. [34] as the baseline for our intended organization. The usage of previously validated devices as the wearable nodes evades the necessity of proposing a novel instrument before developing a cooperative environment. This also reinforces the re-use of verified gear, indicated in Section 2.1.

The analysis of the hardware present in the device allows for the estimation of sensor reading latency. This information has a crucial role in the availability of the data, as the devices behave as IoT nodes, responding to client queries. Thus, the proposed validation test targets the data availability before any device-wise or algorithmic estimation such as data-fusion or machine learning.

The proposed test set analyzes the data availability using a Quality-of-Service formalization for sensor nodes and cooperative wearable devices. This methodical approach allows a quantitative analysis of the system, approaching stronger validation results.

### 4.4. Challenges

In the last two subsections, we summarized the stages presented in this work. In this section, we systematically present the identified challenges in the Requirements, Design, and Validation of CWS. This is a qualitative analysis, which takes into consideration both theoretical and practical phases.

- Requirements:
  - The requirement establishment must follow UD patterns. This is a technical problem, as these design patterns follow a systematic evaluation process;
  - Safety equipment selection follows a systematic approach called AHP, which identify the targeted problem and its factors. The fulfillment of another systematic stage creates an additional technical challenge on the requirement establishment process;
  - CWS provides an extra set of requirements, especially from network and communication according to the target problem specifications. This issue exposes both technical and academic challenges, as working with state-of-the-art techniques and concepts.
- Design:
  - The requirements must precisely describe the proposal needs, since they work as inputs in the design process. This stage reflects on the quality of the presented solution after the validation processes;
  - The development of hardware and software modules must be parallel. Thus, they individually present technical challenges to systems developers;
  - The integration of independently developed Software and Hardware modules is a mandatory step, which needs to provide an expected result. This integration can expose technical flaws that came unnoticed in the previous stages.
- Validation:
  - After the design and development stages, the modules must be properly validated. Thus, this requires the establishment of a theoretical approach or the usage of a previous baseline. Within this context, the solution novelty presents challenges in both practical and theoretical approaches.
  - Many validation processes use prototypes as test mediums. This exposes the cost and time restrictions in the project development. This aspect is critical as both the academic results and the technical validations depend on the employed experimental set.

### 4.5. Final Considerations

The previous analysis validates both the theoretical and practical aspects of this work. The case study links the theoretical analysis to a practical aspect, allowing the observation of the proposed conjectures. Finally, this work fulfills its objects through these aspects—first analyzing the requirement establishment and design process for FR-CWS and then validating these processes through a case-study.

## Figures and Tables

**Table 1:** Sampling time ratio for each sensor.

	Variable	Sensor	Sampling Time
User	Temperature and Humidity	AM2302 (DHT22) [64]	2 s
	IMU	MPU6050 [65]	0.125 ms
	EMG	Myoware	System Sampling Rate [66] (∼5 µs)
	ECG	MAX30100 [67]	1 ms
Environmental	Temperature and Humidity	AM2302 (DHT22) [64]	2 s
	GPS	FGPMMOPA6H [68]	0.1 s
	Luminosity	TSL2561 [69]	2.5 µs

**Table 2:** WPAN and WLAN connectivity technologies.

	Range	Technologies
WPAN	up to 100 m	blacktooth LE, ZigBee, Thread (6LoWPAM), Z-Wave, ANT^+^, WirelessHART, ISA100.11a (6LoWPAM), EnOcean, …
WLAN	up to 1000 m	802.11a/b/n/ac, 802.11af, 802.11ah & 802.11p

## Abbreviations

The following abbreviations are used in this manuscript:

IoT	Internet of Things
HUD	Head-Up Display
HMD	Head-Mounted Display
OS	Operating System
CWS	Cooperative Wearable System
FR-CWS	Field Research Cooperative Wearable Systems
UD	Universal Design
AHP	Analytic Hierarchic Process
WSN	Wireless Sensor Network
WBAN	Wireless Body-Area Network
WPAN	Wireless Personal Area Network
WLAN	Wireless Local Area Network
WNAN	Wireless Neighborhood Area Network
WWAN	Wireless Wide Area Network
IMU	Inertial Measurement Unit
PCB	Printed Circuit Board
CPU	Central Processing Unit
I2C	Inter-IC Communications
QoS	Quality-of-Service
DoF	Degree-of-Freedom
ECG	Electrocardiography
EMG	Electromyography
GPIO	General Purpose Input/Output
MCU	Microcontroller Unit

## References

[1] Soh P.J., Vandenbosch G.A., Mercuri M., Schreurs D.M.P. (2015). Wearable wireless health monitoring: Current developments, challenges, and future trends. IEEE Microw. Mag..

[2] Risling T. (2017). Educating the nurses of 2025: Technology trends of the next decade. Nurse Educ. Pr..

[3] Vogenberg F.R., Santilli J. (2018). Healthcare trends for 2018. Am. Health Drug Benefits.

[4] Liu L., Peng Y., Liu M., Huang Z. (2015). Sensor-based human activity recognition system with a multilayered model using time series shapelets. Knowl. Based Syst..

[5] Zhang Y., Gravina R., Lu H., Villari M., Fortino G. (2018). PEA: Parallel electrocardiogram-based authentication for smart healthcare systems. J. Netw. Comput. Appl..

[6] Qiu S., Wang Z., Zhao H., Liu L., Li J., Jiang Y., Fortino G. (2018). Body sensor network based robust gait analysis: Toward clinical and at home use. IEEE Sens. J..

[7] Pace P., Aloi G., Gravina R., Caliciuri G., Fortino G., Liotta A. (2018). An edge-based architecture to support efficient applications for healthcare industry 4.0. IEEE Trans. Ind. Inform..

[8] Kaya T., Liu G., Ho J., Yelamarthi K., Miller K., Edwards J., Stannard A. (2019). Wearable Sweat Sensors: Background and Current Trends. Electroanalysis.

[9] Camomilla V., Bergamini E., Fantozzi S., Vannozzi G. (2018). Trends supporting the in-field use of wearable inertial sensors for sport performance evaluation: A systematic review. Sensors.

[10] Fu Q.K., Hwang G.J. (2018). Trends in mobile technology-supported collaborative learning: A systematic review of journal publications from 2007 to 2016. Comput. Educ..

[11] Chang C.Y., Lai C.L., Hwang G.J. (2018). Trends and research issues of mobile learning studies in nursing education: A review of academic publications from 1971 to 2016. Comput. Educ..

[12] Kong X.T., Luo H., Huang G.Q., Yang X. (2018). Industrial wearable system: The human-centric empowering technology in Industry 4.0. J. Intell. Manuf..

[13] Rice M., Ma K.T., Tay H.H., Kaliappan J., Koh W.L., Tan W.P., Ng J. Evaluating an augmented remote assistance platform to support industrial applications. Proceedings of the IEEE 4th World Forum on Internet of Things (WF-IoT).

[14] Li Y., khan A., Wang T., Li L., Li C., Yang Y., Liu L. Hand Gesture recognition and real-time game control based on a wearable band with 6-axis sensors. Proceedings of the International Joint Conference on Neural Networks (IJCNN).

[15] Haghi M., Thurow K., Stoll R. (2017). Wearable devices in medical internet of things: scientific research and commercially available devices. Healthc. Inform. Res..

[16] Bhatt C., Dey N., Ashour A.S. (2017). Internet of Things and Big Data Technologies for Next Generation Healthcare.

[17] Constant N., Borthakur D., Abtahi M., Dubey H., Mankodiya K. (2017). Fog-assisted wiot: A smart fog gateway for end-to-end analytics in wearable internet of things. arXiv.

[18] Kong X.T., Yang X., Huang G.Q., Luo H. The impact of industrial wearable system on industry 4.0. Proceedings of the IEEE 15th International Conference on Networking, Sensing and Control (ICNSC).

[19] Kos A., Milutinović V., Umek A. (2019). Challenges in wireless communication for connected sensors and wearable devices used in sport biofeedback applications. Future Gener. Comput. Syst..

[20] Li J., Peng Z., Gao S., Xiao B., Chan H. (2017). Smartphone-assisted energy efficient data communication for wearable devices. Comput. Commun..

[21] Rhodes B.J. (1997). The wearable remembrance agent: A system for augmented memory. Pers. Technol..

[22] Bonato P. (2003). Wearable sensors/systems and their impact on biomedical engineering. IEEE Eng. Med. Biol. Mag..

[23] Billinghurst M., Starner T. (1999). Wearable devices: new ways to manage information. Computer.

[24] Pascoe J. Adding generic contextual capabilities to wearable computers. Proceedings of the 2nd international symposium on wearable computers.

[25] Wei J. (2014). How Wearables Intersect with the Cloud and the Internet of Things: Considerations for the developers of wearables. IEEE Consum. Electron. Mag..

[26] Delabrida S., D’Angelo T., Oliveira R.A.R., Loureiro A.A.F. (2016). Wearable hud for ecological field research applications. Mob. Netw. Appl..

[27] Silva M., Delabrida S., Ribeiro S., Oliveira R. Toward the Design of a Novel Wearable System for Field Research in Ecology. Proceedings of the VIII Brazilian Symposium on Computing Systems Engineering (SBESC).

[28] Silva M.C., Ribeiro S.P., Delabrida S., Oliveira R.A.R. Smart-Helmet development for Ecological Field Research Applications. Proceedings of the XLVI Integrated Software and Hardware Seminar, SBC.

[29] Birenboim A., Dijst M., Scheepers F.E., Poelman M.P., Helbich M. (2019). Wearables and location tracking technologies for mental-state sensing in outdoor environments. Prof. Geogr..

[30] Delabrida S., Billinghurst M., Thomas B.H., Rabelo R.A., Ribeiro S.P. Design of a wearable system for 3D data acquisition and reconstruction for tree climbers. Proceedings of the SA’17 SIGGRAPH Asia 2017 Mobile Graphics and Interactive Applications.

[31] Delabrida S., D’Angelo T., Oliveira R.A., Loureiro A.A. (2016). Building wearables for geology: An operating system approach. ACM Sigops Oper. Syst. Rev..

[32] Chen S.T., Lin S.S., Lan C.W., Hsu H.Y. (2018). Design and Development of a Wearable Device for Heat Stroke Detection. Sensors.

[33] Velázquez R., Pissaloux E., Rodrigo P., Carrasco M., Giannoccaro N., Lay-Ekuakille A. (2018). An outdoor navigation system for blind pedestrians using GPS and tactile-foot feedback. Appl. Sci..

[34] J. P. Amorim V., C. Silva M., A. R. Oliveira R. (2019). Software and Hardware Requirements and Trade-Offs in Operating Systems for Wearables: A Tool to Improve Devices’ Performance. Sensors.

[35] Shapira A., Goldenberg M. (2005). AHP-based equipment selection model for construction projects. J. Constr. Eng. Manag..

[36] Zaneldin E., Sivaloganathan S. A framework for the selection of heavy construction equipment. Proceedings of the American Society for Engineering Management (ASEM) International Annual Conference of the American Society for Engineering Management.

[37] Yılmaz Kaya B., Dağdeviren M. (2016). Selecting occupational safety equipment by MCDM approach considering universal design principles. Hum. Factors Ergon. Manuf. Serv. Ind..

[38] Liang J.M., Su W.C., Chen Y.L., Wu S.L., Chen J.J. (2019). Smart Interactive Education System Based on Wearable Devices. Sensors.

[39] Lim S.M., Oh H.C., Kim J., Lee J., Park J. (2018). LSTM-Guided Coaching Assistant for Table Tennis Practice. Sensors.

[40] Zheng M., Liu P.X., Gravina R., Fortino G. (2018). An Emerging Wearable World: New Gadgetry Produces a Rising Tide of Changes and Challenges. IEEE Syst. Man Cybern. Mag..

[41] Fortino G., Parisi D., Pirrone V., Di Fatta G. (2014). BodyCloud: A SaaS approach for community body sensor networks. Future Gener. Comput. Syst..

[42] Xu M., Qian F., Pushp S. (2017). Enabling cooperative inference of deep learning on wearables and smartphones. arXiv.

[43] Ometov A., Kozyrev D., Rykov V., Andreev S., Gaidamaka Y., Koucheryavy Y. (2017). Reliability-centric analysis of offloaded computation in cooperative wearable applications. Wirel. Commun. Mob. Comput..

[44] Zhang W., Zhang Z., Zeadally S., Chao H.C., Leung V. (2019). MASM: A Multiple-algorithm Service Model for Energy-delay Optimization in Edge Artificial Intelligence. IEEE Trans. Ind. Inform..

[45] Augimeri A., Fortino G., Galzarano S., Gravina R. Collaborative body sensor networks. Proceedings of the IEEE International Conference on Systems, Man, and Cybernetics.

[46] Fortino G., Galzarano S., Gravina R., Li W. (2015). A framework for collaborative computing and multi-sensor data fusion in body sensor networks. Inf. Fusion.

[47] Mihovska A., Sarkar M. (2018). Cooperative Human-Centric Sensing Connectivity.

[48] Zhang X., Yang Z., Chen T., Chen D., Huang M.C. (2019). Cooperative Sensing and Wearable Computing for Sequential Hand Gesture Recognition. IEEE Sens. J..

[49] Peng Y., Peng L. (2016). A cooperative transmission strategy for body-area networks in healthcare systems. IEEE Access.

[50] Khanh N.-H., Song C.G., Lee S.-W. (2018). Smartwatch/Smartphone Cooperative Indoor Lifelogging System. Int. J. Eng. Technol. Innov..

[51] Pimentel G., Rodrigues S., Silva P.A., Vilarinho A., Vaz R., Cunha J.P.S. (2019). A Wearable Approach for Intraoperative Physiological Stress Monitoring of Multiple Cooperative Surgeons. Int. J. Med Inform..

[52] Prakash R., Ganesh A.B. Establishment of network coded cooperative communication for clinical healthcare. Proceedings of the 2nd International Conference on Contemporary Computing and Informatics (IC3I).

[53] Pham M.H., Warmerdam E., Elshehabi M., Schlenstedt C., Bergeest L.M., Heller M., Haertner L., Ferreira J., Berg D., Schmidt G. (2018). Validation of a lower back “wearable”-based sit-to-stand and stand-to-sit algorithm for patients with Parkinson’s disease and older adults in a home-like environment. Front. Neurol..

[54] Sodhro A., Sangaiah A., Sodhro G., Lohano S., Pirbhulal S. (2018). An energy-efficient algorithm for wearable electrocardiogram signal processing in ubiquitous healthcare applications. Sensors.

[55] Firouzi F., Rahmani A.M., Mankodiya K., Badaroglu M., Merrett G.V., Wong P., Farahani B. (2018). Internet-of-Things and big data for smarter healthcare: From device to architecture, applications and analytics. Future Gener. Comput. Syst..

[56] Costa L., Farias R., Santiago A., Silva I., Barros I. (2018). Abiotic factors drives floristic variations of fern’s metacommunity in an Atlantic Forest remnant. Braz. J. Biol..

[57] Arena M.V., Martines M.R., da Silva T.N., Destéfani F.C., Mascotti J.C., Silva-Zacarin E.C., Toppa R.H. (2018). Multiple-scale approach for evaluating the occupation of stingless bees in Atlantic forest patches. For. Ecol. Manag..

[58] Calvão L.B., Juen L., de Oliveira Junior J.M.B., Batista J.D., Júnior P.D.M. (2018). Land use modifies Odonata diversity in streams of the Brazilian Cerrado. J. Insect Conserv..

[59] Pereira A., Nunes F., AICOS F.P. Physical Activity Intensity Monitoring of Hospital Workers using a Wearable Sensor. Proceedings of the 12th EAI International Conference on Pervasive Computing Technologies for Healthcare (PervasiveHealth’18).

[60] Jahanbanifar S., Akhavian R. Evaluation of wearable sensors to quantify construction workers muscle force: an ergonomic analysis. Proceedings of the 2018 Winter Simulation Conference.

[61] Weeks D.L., Sprint G.L., Stilwill V., Meisen-Vehrs A.L., Cook D.J. (2018). Implementing wearable sensors for continuous assessment of daytime heart rate response in inpatient rehabilitation. Telemed. E-Health.

[62] Tjhai C., Steward J., Lichti D., O’Keefe K. Using a mobile range-camera motion capture system to evaluate the performance of integration of multiple low-cost wearable sensors and gait kinematics for pedestrian navigation in realistic environments. Proceedings of the IEEE/ION Position, Location and Navigation Symposium (PLANS).

[63] Kiss F., Boldt R., Pfleging B., Schneegass S. Navigation systems for motorcyclists: exploring wearable tactile feedback for route guidance in the real world. Proceedings of the 2018 CHI Conference on Human Factors in Computing Systems.

[64] AM2302 (DTH22) Datasheet. https://www.sparkfun.com/datasheets/Sensors/Temperature/DHT22.pdf.

[65] MPU-6000 and MPU-6050 Product Specification - Revision 3.4. http://www.invensense.com/wp-content/uploads/2015/02/MPU-6000-Datasheet1.pdf.

[66] BCM2835 ARM Peripherals Datasheet. https://www.raspberrypi.org/documentation/hardware/raspberrypi/bcm2835/BCM2835-ARM-Peripherals.pdf.

[67] MAX30100 - Pulse Oximeter and Heart-Rate Sensor IC for Wearable Health Datasheet. https://datasheets.maximintegrated.com/en/ds/MAX30100.pdf.

[68] FGPMMOPA6H GPS Standalone Module Datasheet. https://cdn-shop.adafruit.com/datasheets/GlobalTop-FGPMMOPA6H-Datasheet-V0A.pdf.

[69] TSL2560, TSL2561 Light-to-Digital Converter Datasheet. https://cdn-shop.adafruit.com/datasheets/TSL2561.pdf.

[70] Waters L.E., Lange R.A. (2015). An updated calibration of the plagioclase-liquid hygrometer-thermometer applicable to basalts through rhyolites. Am. Miner..

[71] Yadav N., Bleakley C. (2016). Fast calibration of a 9-DOF IMU using a 3 DOF position tracker and a semi-random motion sequence. Measurement.

[72] Öberg T. (1995). Muscle fatigue and calibration of EMG measurements. J. Electromyogr. Kinesiol..

[73] Menge F., Seeber G., Volksen C., Wubbena G., Schmitz M. Results of absolute field calibration of GPS antenna PCV. Proceedings of the 11th International Technical Meeting of the Satellite Division of The Institute of Navigation (ION GPS 1998).

[74] Schrama C., Reijn H. (1999). Novel calibration method for filter radiometers. Metrologia.

[75] Hayes M.J., Smith P.R. (2001). A new method for pulse oximetry possessing inherent insensitivity to artifact. IEEE Trans. Biomed. Eng..

[76] Wu F., Redouté J.M., Yuce M.R. (2018). We-safe: A self-powered wearable IoT sensor network for safety applications based on LoRa. IEEE Access.

[77] Varatharajan R., Manogaran G., Priyan M.K., Sundarasekar R. (2018). Wearable sensor devices for early detection of Alzheimer disease using dynamic time warping algorithm. Clust. Comput..

[78] Roopaei M., Rad P., Prevost J.J. A Wearable IoT with Complex Artificial Perception Embedding for Alzheimer Patients. Proceedings of the 2018 World Automation Congress (WAC).

[79] Li B., Dong Q., Downen R.S., Tran N., Jackson J.H., Pillai D., Zaghloul M., Li Z. (2019). A wearable IoT aldehyde sensor for pediatric asthma research and management. Sens. Actuators B Chem..

[80] Nousias S., Tselios C., Bitzas D., Lalos A.S., Moustakas K., Chatzigiannakis I. Uncertainty management for wearable iot wristband sensors using Laplacian-based matrix completion. Proceedings of the 2018 IEEE 23rd International Workshop on Computer Aided Modeling and Design of Communication Links and Networks (CAMAD).

[81] Gia T.N., Sarker V.K., Tcarenko I., Rahmani A.M., Westerlund T., Liljeberg P., Tenhunen H. (2018). Energy efficient wearable sensor node for IoT-based fall detection systems. Microprocess. Microsyst..

[82] Mahmoud M.S., Mohamad A.A. (2016). A study of efficient power consumption wireless communication techniques/modules for internet of things (IoT) applications. Sci. Res..

[83] Boukerche A., Samarah S. (2008). A novel algorithm for mining association rules in wireless ad hoc sensor networks. IEEE Trans. Parallel Distrib. Syst..

[84] Silva M., Oliveira R. Analyzing the Effect of Increased Distribution on a Wearable Appliance. Proceedings of the 2019 IEEE 43rd Annual Computer Software and Applications Conference (COMPSAC).

